# Fibromyalgia Impact Reduction Using Online Personal Health Informatics: Longitudinal Observational Study

**DOI:** 10.2196/15819

**Published:** 2020-04-07

**Authors:** William Collinge, Robert Soltysik, Paul Yarnold

**Affiliations:** 1 Collinge and Associates, Inc Eugene, OR United States; 2 Optimal Data Analysis LLC Glen Burnie, MD United States; 3 Optimal Data Analysis LLC Chicago, IL United States

**Keywords:** fibromyalgia, fibromyalgia impact, health informatics, predictive analytics, symptom reduction, functional status, personalized medicine, health diary

## Abstract

**Background:**

Personal health informatics have the potential to help patients discover personalized health management strategies that influence outcomes. Fibromyalgia (FM) is a complex chronic illness requiring individualized strategies that may be informed by analysis of personal health informatics data. An online health diary program with dynamic feedback was developed to assist patients with FM in identifying symptom management strategies that predict their personal outcomes, and found reduced symptom levels associated with program use.

**Objective:**

The aim of this study was to determine longitudinal associations between program use and functional impact of FM as measured by scores on a standardized assessment instrument, the Fibromyalgia Impact Questionnaire (FIQ).

**Methods:**

Participants were self-identified as diagnosed with FM and recruited via online FM advocacy websites. Participants used an online health diary program (“SMARTLog”) to report symptom ratings, behaviors, and management strategies used. Based on single-subject analysis of the accumulated data over time, individualized recommendations (“SMARTProfile”) were then provided by the automated feedback program. Indices of program use comprised of cumulative numbers of SMARTLogs completed and SMARTProfiles received. Participants included in this analysis met a priori criteria of sufficient program use to generate SMARTProfiles (ie, ≥22 SMARTLogs completed). Users completed the FIQ at baseline and again each subsequent month of program use as follow-up data for analysis. Kendall tau-b, a nonparametric statistic that measures both the strength and direction of an ordinal association between two repeated measured variables, was computed between all included FIQ scores and both indices of program use for each subject at the time of each completed FIQ.

**Results:**

A total of 76 users met the a priori use criteria. The mean baseline FIQ score was 61.6 (SD 14.7). There were 342 FIQ scores generated for longitudinal analysis via Kendall tau-b. Statistically significant inverse associations were found over time between FIQ scores and (1) the cumulative number of SMARTLogs completed (tau-b=–0.135, *P*<.001); and (2) the cumulative number of SMARTProfiles received (tau-b=–0.133, *P*<.001). Users who completed 61 or more SMARTLogs had mean follow-up scores of 49.9 (n=25, 33% of the sample), an 18.9% drop in FM impact. Users who generated 11 or more new SMARTProfiles had mean follow-up scores of 51.8 (n=23, 30% of the sample), a 15.9% drop.

**Conclusions:**

Significant inverse associations were found between FIQ scores and both indices of program use, with FIQ scores declining as use increased. Based on established criteria for rating FM severity, the top one-third of users in terms of use had clinically significant reductions from “severe” to “moderate” FM impact. These findings underscore the value of self-management interventions with low burden, high usability, and high perceived relevance to the user.

**Trial Registration:**

ClinicalTrials.gov NCT02515552; https://clinicaltrials.gov/ct2/show/NCT02515552

## Introduction

Health informatics is a promising field that includes the use of internet technology to create new models of patient care and disease management by analyses of large bodies of health-related data. Health informatics are traditionally associated with large population databases (eg, the insurance industry or “big data”); however, patient-level interventions that employ health informatics also have the potential to empower individuals to take greater control over their symptoms and outcomes [1]. Combining personal health informatics with emerging techniques of predictive analytics represents an intriguing and potentially powerful approach to personalized medicine for some of the most confounding health conditions.

Such innovative strategies may be particularly helpful in complex, multisystemic chronic illnesses that have inconsistent or unreliable treatment responses across patients, such as in conditions where no effective treatments are available or where complementary therapies, drugs, dosages, or self-care strategies have widely varying effects across different individuals with the same diagnosis. Fibromyalgia (FM) is one such condition in which optimal symptom management calls for highly individualized integration of pharmacological and nonpharmacological approaches [2].

We have developed an electronic health (eHealth) intervention consisting of a daily health diary designed to enhance an individual’s awareness of health-related actions and their impacts in daily living. In addition to the heightened self-awareness that may be anticipated from regular use of any such diary, the program incorporates a predictive analytics algorithm that processes the user’s accumulated data to produce personalized intervention guidance driven by those data. We previously reported data on effects of the program (now revised and called AwareHealth) on *symptom ratings* in users with FM. Use was found associated with significant reductions in pain, stiffness, fatigue, concentration problems, memory problems, feeling anxious, feeling depressed, gastrointestinal problems, and sleep difficulties [3].

This paper reports the effects of program use on the *functional impact* of FM as defined by the Fibromyalgia Impact Questionnaire (FIQ), a standardized instrument used widely in FM clinical outcome research [4]. The FIQ was developed to measure the status, progress, and outcomes of patients with FM by measuring the components of health status believed to be most affected by FM using a 1-week recall period. The FIQ contains 11 questions related to physical functioning, each rated on a 4-point Likert type scale; two items asking the patient to mark the number of days they felt well and the number of days they were unable to work (including housework) because of FM symptoms; and seven horizontal linear scales, marked in 10 increments, on which the patient rates work difficulty, pain, fatigue, morning tiredness, stiffness, anxiety, and depression. Scoring provides a global measure of FM impact from 0 (lowest impact) to 100 (highest impact).

## Methods

### Subjects and Recruitment

In an online study sponsored by the US National Institute of Arthritis, Musculoskeletal and Skin Diseases [5], participants were recruited via public announcements on several FM advocacy websites. Enrollment and consent were processed online with electronic attestation of being 18 years or older and having been diagnosed with FM by a health professional. No financial or other incentive was offered. Human participant oversight was provided by the New England Institutional Review Board.

### Intervention

The intervention program consisted of a brief health diary (“SMARTLog”) completed by the user in about 5 minutes, three or more times per week over several weeks [6]. For a 24-hour recall period, users entered symptom ratings. They then used drop-down menus and radio buttons to report on four domains of behavioral and lifestyle variables informed by research, theory, and clinical observation as potentially influencing symptoms in FM, and a fifth domain for tracking user-defined inputs such as pharmacological or nonpharmacological therapies, or other variables of interest to the individual such as foods or activities. Textbox 1 shows the SMARTLog contents of the original version, which was used for the data reported in this paper. The revised version, which is in current use, provides an expanded array of 75 user-selectable symptoms and wellness outcomes, and adds a new domain, “Technology use”, to report duration of screen time (eg, TV, movie, computer, mobile device) and wireless device exposure (see Multimedia Appendix 1).

**SMARTLog Contents (Original Version).**

**Outcomes**

Pain, stiffness, fatigue, concentration problems, memory problems, feeling anxious, feeling depressed, gastrointestinal problems, and sleep difficulties, all rated on a 0-10 scale for “how bothersome” for the 24-hour recall period

**Sleep and rest**

Time to bed the previous night, wake-up time, and times and durations of daytime naps

**Meals and snacks**

Time and size of meals and snacks

**Self-care practices**

Time and duration of bathing; mind, body, or spirit practices; and exercise including the type, duration, and exertion level

**Daily activities**

Duration, exertion level, satisfaction level, and stressfulness of the following types of activities: vocational, domestic activity, social activity, recreation, travel or commuting time, and the day’s overall activity level

**Personal inputs (optional)**

Users may self-define up to ten additional inputs to track (eg, specific medications, complementary therapies, dosages, foods, nutritional supplements, or activities not listed earlier), with user-selected metrics for each, such as quantities or durations.

The data from SMARTLog accumulates in the user’s personal database and are processed daily in the background by a proprietary automated algorithm using single-subject (N-of-1) analytic methods to find statistically significant (alpha=.05 level) associations between symptom levels and specific health-related actions. This algorithm applies optimal discriminant analysis, a nonparametric method for maximizing classification accuracy in models with two classes and an ordered attribute (independent variable) [7]. The likelihood of finding significant associations increases as the volume of data accumulates. Users are encouraged to experiment by employing various strategies (eg, different bedtimes, dosages of a drug, complementary therapies, durations of exercise), as variability of data helps the program discover models that predict symptom reduction. When associations are found, the algorithm uses a natural language generator to report them to the user as “SMARTProfile” statements (eg, “My pain will improve if my bed time is no later than 9:40 pm”, “My fatigue will improve if my dosage of Lyrica is no more than 50 mg”, or “My sleep will improve if my yoga is at least 20 minutes”). See Multimedia Appendix 1.

### Data Collection

All participation and data collection was online via the project website. Participants completed an FIQ at baseline, and then every subsequent 30 days of program use as “follow-ups” during an 11-month open-use period. Users were free to use the SMARTLog program as often and for as many or as few months as they wished during the study period. In addition to the monthly FIQ, cumulative counts of the number of SMARTLogs and SMARTProfiles accruing for each subject were used for analysis.

### Data Analysis

To determine longitudinal associations between FIQ follow-up scores and program use we used Kendall tau-b, a nonparametric statistic that measures both the strength and direction of an ordinal association between two repeated measured variables [8]. We computed this between all FIQ scores and the *indices of program use* for each subject in the sample (ie, the cumulative numbers of SMARTLogs and SMARTProfiles produced for each subject at the time of each completed FIQ).

Since the use data were skewed, with relatively few users showing high rates of use compared to others, Kendall tau-b was more appropriate for these data than the more commonly used Pearson correlation *r*, as the latter inflates Type I error rates and reduces power when the data are not normally distributed [9]. Kendall tau-b was also preferred to the Spearman rank correlation coefficient *r_S_*, as tau-b exhibits improved performance in the presence of contaminated normal distributions with outliers [10] and has better statistical properties; as explained by Howell, “Kendall’s *τ* has generally been given preference of Spearman’s *r_S_* because it is a better estimate of the corresponding population parameter, and its standard error is known,” [11]. It was also felt that this statistic better characterized the individualized analysis in this case.

All associations were 2-tailed and evaluated at the alpha=.05 level of statistical significance. Prior studies found no demographic predictors of study participation vs nonparticipation. Software used was JMP statistical software (version 14.0).

## Results

### Sample

As is common in remote online studies, applicants varied widely in their levels of commitment to participate, with many applying out of curiosity. This analysis focuses on users who had sufficient levels of program use over the 11-month open-access study period to activate the analytic functionality provided by the SMARTProfile feature of the program. In pilot testing we determined that at least 22 completed SMARTLogs were needed to generate at least 1 SMARTProfile statement [12]. Thus, we set 22 or more SMARTLogs as the a priori criterion for selecting the sample for this analysis.

**Figure 1:**
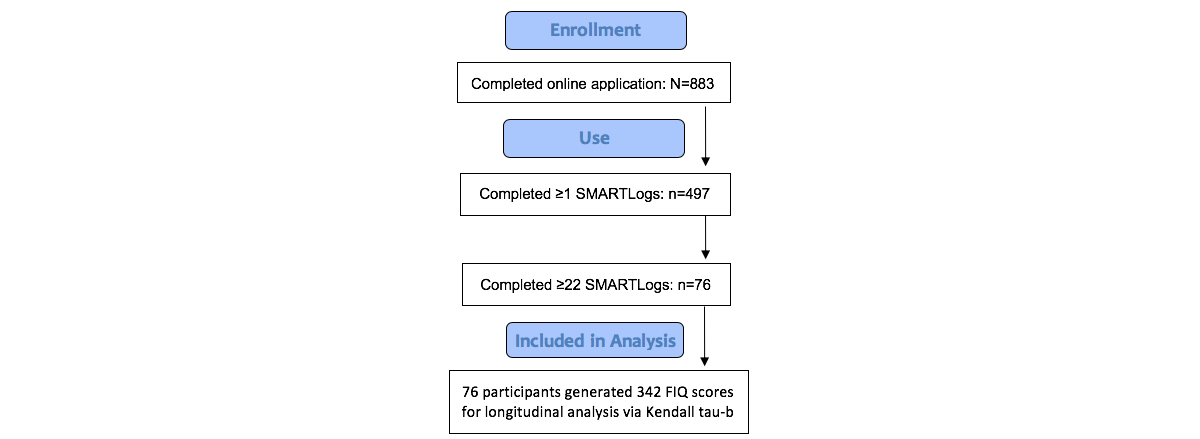
CONSORT (Consolidated Standards of Reporting Trials) flow diagram. FIQ: Fibromyalgia Impact Questionnaire.

Of 497 study applicants completing at least 1 SMARTLog, a sample of 76 participants met the use criteria for this analysis Figure 1. Of these, we classified those who completed 22 to 47 SMARTLogs (n=46) as moderate users, and ≥48 SMARTLogs (n=30) as heavy users. We found no demographic predictors of use. Moderate users completed a mean of 32.9 (SD 6.6) SMARTLogs, and heavy users completed a mean of 95.3 (SD 52.5) SMARTLogs. The mean number of follow-up FIQs provided by moderate users (n=46) was 2.54 (SD 1.46), and for heavy users (n=30), 4.97 (SD 2.20).

Of the 76 participants, 99% (75) were female and 96% (73) were white, with a mean age of 47 (SD 12) years. About 70% (53) were college graduates, 32% (24) described themselves as disabled, 43% (33) were working part or full time, 5% (4) were seeking employment, and 20% (15) were retired. Mean reported duration of being diagnosed with FM was 13.6 (SD 10.8) years. The most commonly reported concurrent medical conditions were osteoarthritis (26, 34%), hypertension (22, 29%), chronic fatigue syndrome (21, 28%), and gastroesophageal problems (17, 22%). The number of monthly follow-up FIQs available for analysis per subject ranged from 1-9.

### Change in FIQ scores

The sample had a mean baseline FIQ score of 61.6 (SD 14.7, n=76) and provided a total of 342 FIQ scores for longitudinal analysis via Kendall tau-b. As seen in Table 1, statistically significant inverse correlations were found over time between FIQ scores and both indices of program use (ie, as SMARTLogs or SMARTProfiles accumulated, FM impact was reduced).

Users who completed 61 or more SMARTLogs had mean follow-up scores of 49.9 (25/76, 33% of the sample), an 18.9% drop in FM impact. Users who generated 11 or more new SMARTProfiles had mean follow-up scores of 51.8 (23/76, 30% of the sample), a 15.9% drop.

**Table 1:** Longitudinal associations of program use with FIQ scores (n=76).

Index of use	Tau-b	*P* value
Cumulative number of SMARTLogs completed	–0.135	<.001
Cumulative number of SMARTProfiles received	–0.133	<.001

## Discussion

### Principal Results

Statistically significant longitudinal associations were found between both indices of use and FIQ scores; specifically, as cumulative numbers of each index for a user increased, FIQ scores declined, indicating reduced functional impact of FM. In terms of duration of program use, we noted that these indices function as proxies of time*,* as users could accumulate a given number of SMARTLogs or SMARTProfiles over variable lengths of time. For example, the fastest a user could accumulate 50 SMARTLogs is 50 days, but the accumulated SMARTLogs could also be spread over 6 months or longer; the statistical model does not treat time duration per se as a variable.

### Clinical Relevance

As a frame of reference for clinical relevance of these associations, we refer to a pooled analysis by Bennett et al [13] of 2228 patients with FM participating in three clinical trials. In that analysis, participants had a mean baseline FIQ score of 62; across the three studies, the estimated minimal clinically important difference using a 95% confidence interval was found to cause a 14% drop in FIQ scores, which the investigators defined as a “clinically relevant outcome”. In their severity analysis, an FIQ total score ≤38 was found to represent “mild” FM impact, 39-58 a “moderate” FM impact, and 59-100 a “severe” FM impact.

Thus, applying the Bennett et al [13] criteria to the present sample, roughly the top one-third of users in terms of frequency of use experienced mean reductions in FM impact levels, moving their scores from the “severe” category to the “moderate” category.

### Limitations

In any self-directed behavioral health intervention, compliance and use are important contributors to positive outcomes. For such interventions to be successful, they must have high usability and high relevance to the user [14]. In this study, the ratio of users who met the a priori use criteria vs total number of project enrollees (76/497, 15.3%) indicates that a relatively high level of use may be required to achieve the greatest clinical benefit from the program.

We do not know what individual participant variables predicted use levels that were sufficient to satisfy our a priori criteria and be included in this analysis. It is possible that limitations imposed by the illness itself are an obstacle to use for many users. Further exploration is needed to determine how a higher level of compliance and use could be encouraged in this patient population.

### Conclusions

The functional impacts of FM in daily living are a significant source of distress. Interventions that aid discovery of personalized strategies to reduce these impacts can make an important contribution to quality of life and well-being in this population, as well as for others with complex chronic illnesses. The findings reported here suggest that clinically significant functional improvement—along with reduced symptoms as previously reported—is possible through an eHealth intervention using personal health informatics. Future research will seek to identify additional predictors of outcomes of the AwareHealth program and explore its use with other chronic conditions.

## Appendix

Multimedia Appendix 1 Using AwareHealth: Quick Tour (video). This video with narration shows the main screens of the AwareHealth program and navigates through the various program functions.

## Abbreviations

eHealth: electronic health
FIQ: Fibromyalgia Impact Questionnaire
FM: fibromyalgia
